# Critical appraisal of RCTs by 3rd year undergraduates after short courses in EBM compared to expert appraisal

**DOI:** 10.3205/zma001171

**Published:** 2018-05-15

**Authors:** B. Buchberger, J.T. Mattivi, C. Schwenke, C. Katzer, H. Huppertz, J. Wasem

**Affiliations:** 1 University of Duisburg-Essen, Faculty of Economics and Business Administration, Institute for Health Care Management and Research, Essen, Germany; 2 SCO:SSiS, Schwenke Consulting: Strategies and Solutions in Statistics, Berlin, Germany

**Keywords:** Critical appraisal, evidence-based medicine, randomized controlled trial, training, undergraduates

## Abstract

**Introduction: **An essential aim of courses in evidence-based medicine (EBM) is to improve the skills for reading and interpreting medical literature adequately. Regarding the conceptual framework, it is important to consider different educational levels.

**Aim: **Our primary aim was to investigate the applicability of different instruments for the assessment of methodological study quality by 3rd grade students after short courses in EBM. Our secondary outcomes were agreement with expert assessments and student’s knowledge and competences.

**Methods: **We conducted four short courses in EBM of 90 minutes each for health care management and medical students focused on critical appraisal of the literature. At the end, the students assessed five publications about randomized controlled trials (RCTs) using five different instruments; the results were compared to expert assessments.

**Results:** In total, 167 students participated in our EBM courses. Students’ assessments showed a non-systematic over- and underestimation of risk of bias compared to expert assessments with no clear direction. Agreement with expert assessments ranged between 66% to over 80%. Across RCTs, evidence was found that the choice of instrument had an impact on agreement rates between expert and student assessments (p=0.0158). Three RCTs showed an influence of the instrument on the agreement rate (p<0.05 each).

**Discussion: **Our results contrast sharply with those of many other comparable evaluations. Reasons may be a lack of students’ motivation due to the compulsory courses, and the comparison to a reference standard in addition to self-ratings causing objectivity.

**Conclusion:** Undergraduates should become familiar with the principles of EBM, including research methods, and the reading of scientific papers as soon as possible. For a deeper understanding, clinical experience seems to be an indispensable precondition. Based on our results, we would recommend an integration of lectures about EBM and critical appraisal at least twice during studies and with greater intensity shortly before graduation.

## Zusammenfassung

**Einleitung:** Ein wesentliches Ziel von Kursen in evidenzbasierter Medizin (EBM) ist die Verbesserung von Fähigkeiten für adäquates Lesen und Interpretieren medizinischer Fachliteratur. Hinsichtlich der Konzeption ist es wichtig, unterschiedliche Ausbildungsgrade zu berücksichtigen.

**Ziel: **Primäres Ziel war die Untersuchung der Anwendbarkeit verschiedener Instrumente zur Bewertung der methodischen Qualität von publizierten Studien durch Studenten im dritten Studienjahr nach einem Kurzlehrgang in EBM. Sekundäre Ziele waren Übereinstimmung mit Experten-Bewertungen sowie Kenntnisse und Kompetenzen der Studenten.

**Methoden: **Vier Veranstaltungen zur EBM von jeweils 90 Minuten wurden für Medizinmanagement- und Medizinstudenten mit dem Fokus auf die kritische Bewertung von Literatur in Hinblick auf ein Bias-Risiko durchgeführt. Am Ende bewerteten die Studenten fünf Publikationen über randomisierte klinische Studien (RCTs) mit fünf verschiedenen Instrumenten; die Ergebnisse wurden mit Bewertungen von Experten verglichen.

**Ergebnisse:** Insgesamt nahmen 167 Studenten an den EBM-Kursen teil. Im Vergleich zu den Experten-Bewertungen zeigten die Bewertungen der Studenten eine nicht systematische Über- und Unterschätzung des Bias-Risikos ohne erkennbare Richtung. Die Übereinstimmung mit den Experten-Bewertungen reichte von 66% bis über 80%. Für die RCTs zeigte sich, dass die Auswahl des Instruments einen Einfluss auf die Übereinstimmungsrate zwischen studentischen und Experten-Bewertungen hatte (p=0,0158). Bei drei RCTs konnte ein Einfluss des Instruments auf die Übereinstimmungsrate festgestellt werden (jeweils p<0,05).

**Diskussion:** Die Ergebnisse stehen in starkem Widerspruch zu denen vieler anderer, vergleichbarer Erhebungen. Gründe können in der mangelnden Motivation der Studenten für die Pflichtveranstaltung liegen. Es ist aber auch möglich, dass der Vergleich mit einem Referenzstandard als Ergänzung zu Selbstauskünften zu Objektivität führte.

**Schlussfolgerung:** Nicht graduierte Studenten sollten mit den Prinzipien der EBM so früh wie möglich vertraut gemacht werden, ebenso mit Forschungsmethoden und der Lektüre von wissenschaftlichen Publikationen. Für ein tieferes Verständnis scheint klinische Erfahrung eine unverzichtbare Voraussetzung zu sein. Auf der Basis unserer Ergebnisse empfehlen wir, Veranstaltungen zur EBM und kritischen Bewertung mindestens zweimal im Studienverlauf und mit größerer Intensität kurz vor dem Abschluss durchzuführen.

## 1. Introduction

The level of awareness towards evidence-based medicine (EBM) is growing worldwide and the acceptance of its concept is increasing. In January 2007 the British Medical Journal conducted an online poll about the 15 most important medical milestones and EBM was in seventh place, right behind germ theory and oral contraceptive pill but ahead of computer and medical imaging [1], [2]. However, fostering an EBM culture and implementing it into practice requires the skills for identifying and appraising the literature critically [3], [4], [5]. A certain knowledge of probability and statistics is mandatory as well when accessing guidelines and evidence summaries, assessing marketing and advertising material from industry, interpreting the results of a screening test, or reading research publications for staying up to date with newly developed treatments; furthermore, knowledge of biostatistics is necessary for the analysis of numerical data, for informing patients about treatment risks, and last but not least for being prepared to the Internet-literature of varying quality presented by patients [6], [7]. Actually, the question is no longer whether to teach EBM but how to teach it [8] and when. Apart from various educational methods, e.g. on the job training, problem-based or self-directed learning [9], the EBM concept may be taught as a whole as well as some of the five steps separately, which are

1. asking a clinical question,
2. searching for the best evidence,
3. critical appraisal of the evidence,
4. applying evidence to patience,
5. self-assessment [8].

There are quite a few courses introducing into database searches supported by librarians [9], or clubs as a format of training critical appraisal of the literature [9], [10], [11]. For measuring the increase of learners‘ competency by attending lectures in EBM, objective measurements are required rather than self-ratings leading to considerable overestimation [12], [13].

Previous studies evaluated the impact of EBM lectures mostly by self-reports of participants [4], [11] or kinds of question papers with multiple-choice questions [14]. In addition to self-assessments, the current study aims to achieve a certain objectivity by comparing students’ assessments with a reference standard created by expert assessments.

Our primary outcome was to investigate the applicability of different instruments for the assessment of methodological study quality by 3rd grade students after short courses in EBM. Our secondary outcomes were agreement with expert assessments and student’s knowledge and competences.

## 2. Methods

We included medical students directly after the preclinical phase at the faculty of medicine and students of the master’s program “health care management” at the faculty of economic sciences of the University of Duisburg-Essen, Germany. They were trained in the principles of EBM in four sessions of 90 minutes each which were held in the context of lectures on health economics (compulsory courses for medical students) by the Institute of Health Care Management and Research in the winter semester of 2013/2014. The number of participants was limited to 20 students per session. A glossary of terms for quick searches was handed out (see Attachment 1).

### 2.1. Description of instruments and experts

Based on a Health Technology Assessment Report, we focused on generic component instruments published after the year 2000 to assess the quality of evidence [15]. A component instrument is a tool to assess all aspects which may introduce bias like randomization or type of blinding. Five instruments were used [16], [17], [18], [19], [20] which differed by the number of domains, questions within domains, and answer options within a question (see Table 1 (Tab. 1)). 

In a second step, the students were asked to rate the overall risk of bias on a five point Likert scale (very low, low, moderate, high, very high), based on the outcomes of the risk assessment. Each student was asked to evaluate publications of five randomized controlled trials (RCTs). Assessing five RCTs by five instruments would lead to 25 combinations of RCTs and instruments. To reduce the efforts for the students, each RCT was assessed by one instrument only, which was randomly selected, resulting in five assessments per student. Permutation was used to ensure that each RCT was assessed with each instrument by the same number of students. 

Then, five experts assessed the five RCTs with a single instrument, which was selected randomly (see Table 2 (Tab. 2) for assignment of instrument and RCT to expert). These assessments were used as the reference standard (‘gold standard’). Experts had to fullfil the following criteria: independence from the University of Duisburg-Essen, experience in critically appraising clinical studies for more than 10 years, and professional status including responsibility for assessments. 

#### 2.2. Training sessions

At the beginning, we presented the concept of EBM and its five steps by means of a specific clinical case and in detail. Essential terms were explained theoretically and in a traditional teaching approach: internal and external validity, quality parameters like randomization, concealment, blinding, drop-out/lost to follow-up, intention-to-treat (ITT) analysis, evidence levels of study designs, PICO-scheme and different kinds of bias. In addition, the structure of scientific publications was explained and therein text passages were indicated, where the description or discussion of validity aspects is most likely to be found. The students did a practical exercise, based on this information, afterwards. As an aid, a slide was shown containing the glossary of terms for quick searches (see Attachment 1).

The second session started with a repetition and consolidation of the knowledge gained. For this purpose, the participants were asked to split into groups and to allocate key words to quality parameters. After internal discussion, the groups used a flip-chart for poster presentation, during which the assignment of the key words to a quality aspect had to be explained to the others. For a further understanding, a simulation of randomization, blinding, concealment, stratification, drop-out/lost to follow-up and different types of analyses (e.g. ITT) was carried out thereafter. 

In the third session, the component systems for the quality assessment were introduced and the single questions of the instruments were discussed. The participants then applied the systems for quasi randomly selected RCTs about the most frequent chronic diseases defined by the WHO, and results were discussed.

In the final session, the students’ skills were tested by application of the component systems on another set of currently published RCT. Again, permutation was done for assigning instruments to RCTs. Analyses of the methodological study are based on these assessments.

#### 2.3. Analysis

Frequencies of assessments given by medical students and health care management students were computed by RCT and instrument. In addition, agreement between student rating and the expert's assessment was defined as “agreement +/-1”: Agreement was considered as attained when the student rating was within a range of +/-1 point of the expert's assessment. Generalized estimation equations were used to investigate the influence of the instrument, the student group and the RCT on the agreement rate in a first analysis. In a second analysis, the effect of instrument and student group was assessed by RCT. In addition, influences of “experience in critically appraising” and “command of English” were investigated. Analyses were performed with SAS 9.2 (SAS Institute Inc., Cary, NC, USA).

## 3. Results

In total, 167 students took part in our EBM courses, of whom 142 were third year undergraduate medical students and 25 students of the master’s program “health care management” (see Table 3 (Tab. 3)).

The investigation of our primary outcome, the applicability of different instruments for the assessment of methodological study quality, did not provide evidence on the comprehensibility of instruments, instructions for using the instruments, if available, or duration of assessment procedure. 

**Figure 1:**
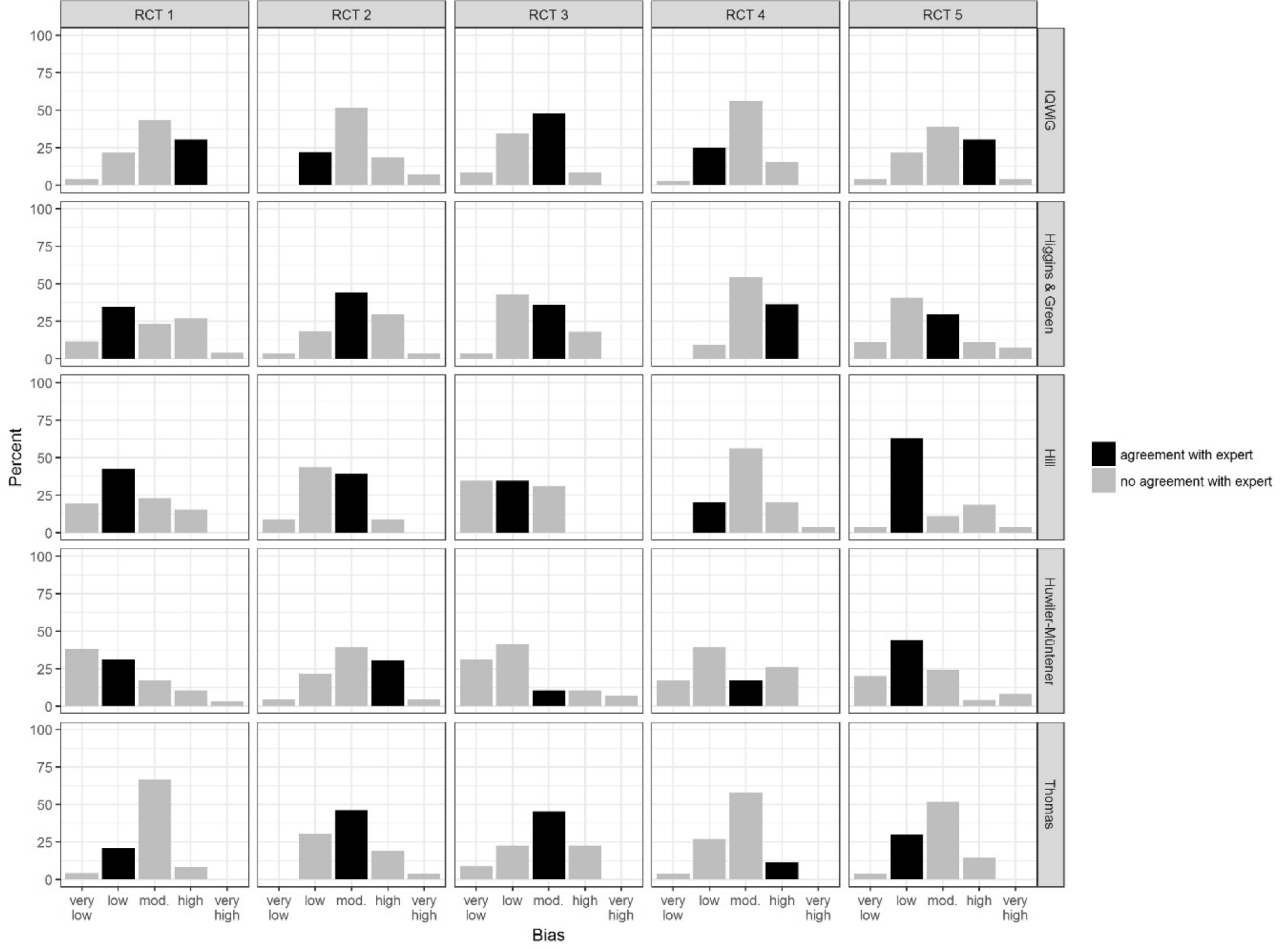
Assessments of medical students by RCT and instrument. Bars represent percentages. RCT 1 [21], RCT 2 [22], RCT 3 [27], RCT 4 [23], RCT 5 [34]

With regard to our secondary outcomes, we only report the significant findings on agreement with expert assessments. Figure 1 (Fig. 1) shows the percentages of medical students, who have given the five ratings by RCT and instrument. The rating with a black bar indicates the expert rating, e.g. for RCT 1 [21] and the IQWiG instrument, the expert assessed the study as having a high potential for bias, for RCT 2 [22], the expert found a low potential for bias, when using the IQWiG instrument. The height of the bar represents the proportion of students by rating in percent. The size of the black bar indicates, how many students got the same rate as the expert. The assessments of the same study by different experts with different instruments however show some variability. To reflect this, the agreement rate was assessed by comparing the students' assessments with a tolerance of +/-1 with the expert assessments. The majority of assessments shows an agreement in a range of 66% to over 80% and therefore an adequate rate. 

Table 4 (Tab. 4) shows the agreement rates for “agreement +/-1” attained by student group in the various RCTs for the five instruments under investigation. Across RCTs, evidence was found that the choice of instrument had an impact (p=0.0158), while no difference for an influence of student group or RCT was found (p=0.3856 and p=0.2425, respectively). By RCT, evidence was found for an influence of the instrument on the agreement rate in RCTs 1, 2 and 3 (p=0.0146, p=0.0263 and p<0.0001, respectively). For the endpoint “agreement +/-1”, no evidence for an influence of “experience in critically appraising” or command of English was found. To note, the description of questions showed very different levels of detail in the publications, e.g. “the allocation code was concealed in sequentially numbered, opaque, sealed envelopes” [21] versus a simple mentioning of the term “randomization” without description of methodological details [23].

## 4. Discussion

We found a non-systematic over- and underestimation of risk of bias compared to the experts' assessments with no clear direction in the students’ assessments, what corresponds somehow to the answers given after the four short courses in EBM: 73% of the medical students rated their knowledge gained as weak or low. Our results contrast sharply with those of many other evaluations after lessons in EBM for undergraduates. In a before-after comparison, Weberschock et al. [14] observed a significant increase of performance in 124 year 3 medical undergraduates in Germany from a score of 2.37 points before the seminar and 9.85 points thereafter (99% CI [8.94; 10.77], p<0.001). Carrying out a controlled educational study, Ghali et al. [11] showed a significant difference in literature searching (p<0.002) and critical appraisal skills (p<0.0002) between third year medical students in Boston visiting either four sessions in EBM or receiving traditional didactic teaching in various clinical topics. A systematic review [4] about the impact of teaching critical appraisal skills including 10 clinical studies as well as a recently published review [9] including 14 RCTs about methods of teaching medical trainees EBM concluded an increase of learner competencies post-intervention across all studies. One reason for these differences compared to our results may be self-selection of highly motivated participants in contrary to our students visiting compulsory courses. In addition, the objectivity of evaluations wasn’t always strong, ranging between full self-perceptions, kinds of question papers with multiple-choice questions [14], and validated tests. As stated by Fritsche et al. 2002 [24] comparing the effects of EBM lectures between experts, postgraduate physicians and medical students, an objective evaluation of courses in EBM may be difficult but essential because there is a poor correlation between subjective perception of knowledge and its objective assessment [3]. 

In our study, students’ assessments were compared to a reference standard created by experts and therefore guaranteeing a certain objectivity, which is always prone to individual experience and individual perception; supplementary self-ratings of an increase in knowledge were recorded. 

As each expert has rated each study by one instrument only, no assessment of reliability of experts was performed. It has to be mentioned here that even most of the existing assessment tools are not tested for validity and reliability [25]. With the exception of two [16], [20] this also applies for the included assessment instruments. Evaluating the Cochrane Collaboration’s risk of bias tool [16], Hartling et al. [26] found a wide range of inter-rater reliability between experts on individual domains from slight to substantial (weighted κ=0.13-0.74). Working in the same institution and review team, the authors assume a much higher variability across different research units. As an explanation for the wide range of inter-rater agreement, the authors discuss the need for clear and detailed instructions to improve reliability [26].

As seen in figure 1 (Fig. 1), the different instruments led the different experts to different assessements of the same study. One reason may be the different domains, additional information asked by some intruments as well as different depth of questions. This may lead to discordant conclusions, if certain aspects are not asked for in some instruments but asked for others. To cover the uncertainty, we used a less strict definition of agreement in terms, that an assessment with +/- one point is still regarded an agreement. Selection bias in the shape of publication bias and reporting bias can be assumed for all of the publications mentioned above which reported clearly positive effects of EBM lectures.

The extent and comprehensibility to which single questions were described within publications varied widely, sometimes leading to an impossible task for our unexperienced undergraduate students and a distortion in the analyses as well. It seems striking that the description of questions published in higher ranked journals (RCT 3 and 4 [27], [23]) requiring compliance with special statements concerning reporting quality was less understandable for our students than in publications with a smaller impact. The fact that critical appraisal always includes subjectivity by interpretation as well as scoring systems which only appear to be objective due to an explicit or implicit weighting without any empirical basis [28] is worth pointing out within this context.

The number of five different assessment instruments could have been too complex. On the other hand, the repetition of terms served for a greater familiarity, and we only addressed step 3 of the EBM concept in detail, therefore focusing the knowledge transfer very strongly. In particular, the training of step 1, formulating a research question which can be operationalized, may take a substantial amount of time for undergraduate students without experience in scientific work. This also applies to step 2, the literature searches in electronic databases.

Teaching critical appraisal separately, as we did, is very common and also known as journal club, meaning that participants have to read and appraise articles critically under the guidance of an expert [4]. For the reason of keeping up to date with new evidence, clinicians have to go through many articles in every day practice, and to do this effectively, training is necessary [29]. Therefore, and as suggested as a format of teaching EBM under certain conditions [10] we focused on step three of the EBM concept. However, this evokes other difficulties as critical appraisal integrates knowledge about epidemiology, information science and biostatistics [9]. 

Although lectures in epidemiology and psychology including statistics are compulsory in the first two years in Germany, 69% of our medical students reported a weak or low knowledge in statistics and/or epidemiology, showing that attitude and knowledge are not spread in the same manner, and teaching EBM must address the needs of different learners [8]. Maybe, using step 1 and 2 of the concept as an introduction is more appropriate to foster a scientific mindset. Alternatively and in order to escape the charge of isolation from clinical practice [30], teaching step 4, applying the evidence on individual cases, may be considered. However, this cannot succeed in undergraduates when practical experience is lacking, and the concrete objective only a vague idea. This is underlined by long-term experiences from Duke and Stanford resulting in a curriculum with the precondition of a clinical training prior to research experience because students were better prepared to understand the clinical and translational potential of their research projects [31]. For an open mind and a better assessment it would also be beneficial if students were familiar with the whole development process of a clinical trial and the impact of its single aspects [32].

In order to get and apply the best available evidence to clinical decision making, skills in finding and critically appraising medical literature are an essential prerequisite [13]. Without background knowledge in methodology and statistics, physicians are at a high risk of misinterpreting evidence, leading to medical errors and adverse effects [28].

Swift et al. [6] investigated the views of 130 physicians about training in statistics and its need in daily practice. As a student, more than half of the participants (60%) underestimated the value of these subjects as relevant to medical practice whilst the majority (73%) had realized its impact for their career over time. Despite the increasing conviction in the relevance of EBM there is evidence for continuing knowledge gaps in basic statistical concepts among practicing physicians and medical researchers [32]. Likewise, a sound of knowledge of key methodological EBM terms and sources seems to be lacking among the majority of health personal including physicians, translational researchers, nurses and other health professionals [12], [13]. To remedy this situation, even students must be helped to perceive these subjects as important to clinical practice [32]. It’s hoped that future physicians will better appraise research findings and contribute to furthering the clinical field by conducting research [33].

## Acknowledgement

We thank Tobias Goeke, Angelika Gohlke, Anja Hagen, Beate Lux, and Monika Nothacker for their expert assessments.

## Ethical approval

The seminar content and structure was approved by the office of the Dean. There was no contact with patients.

## Competing interests

The authors declare that they have no competing interests. 

## Supplementary Material

Glossary of terms for quick searches

## Figures and Tables

**Table 1:**
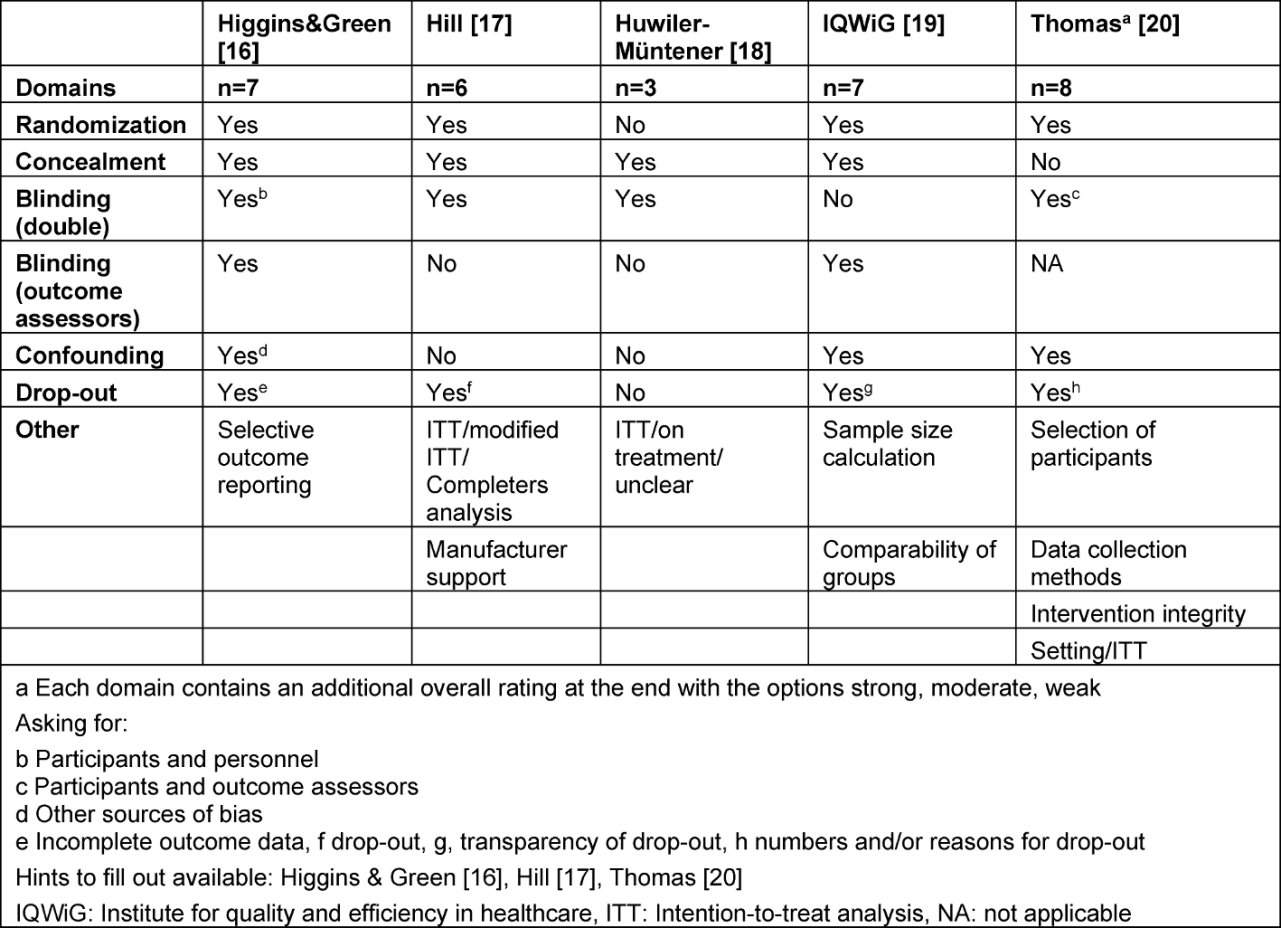
Component Instruments

**Table 2:**
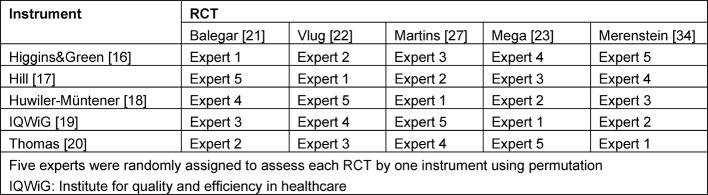
Assignment of Assessment Instrument and RCT to Experts

**Table 3:**
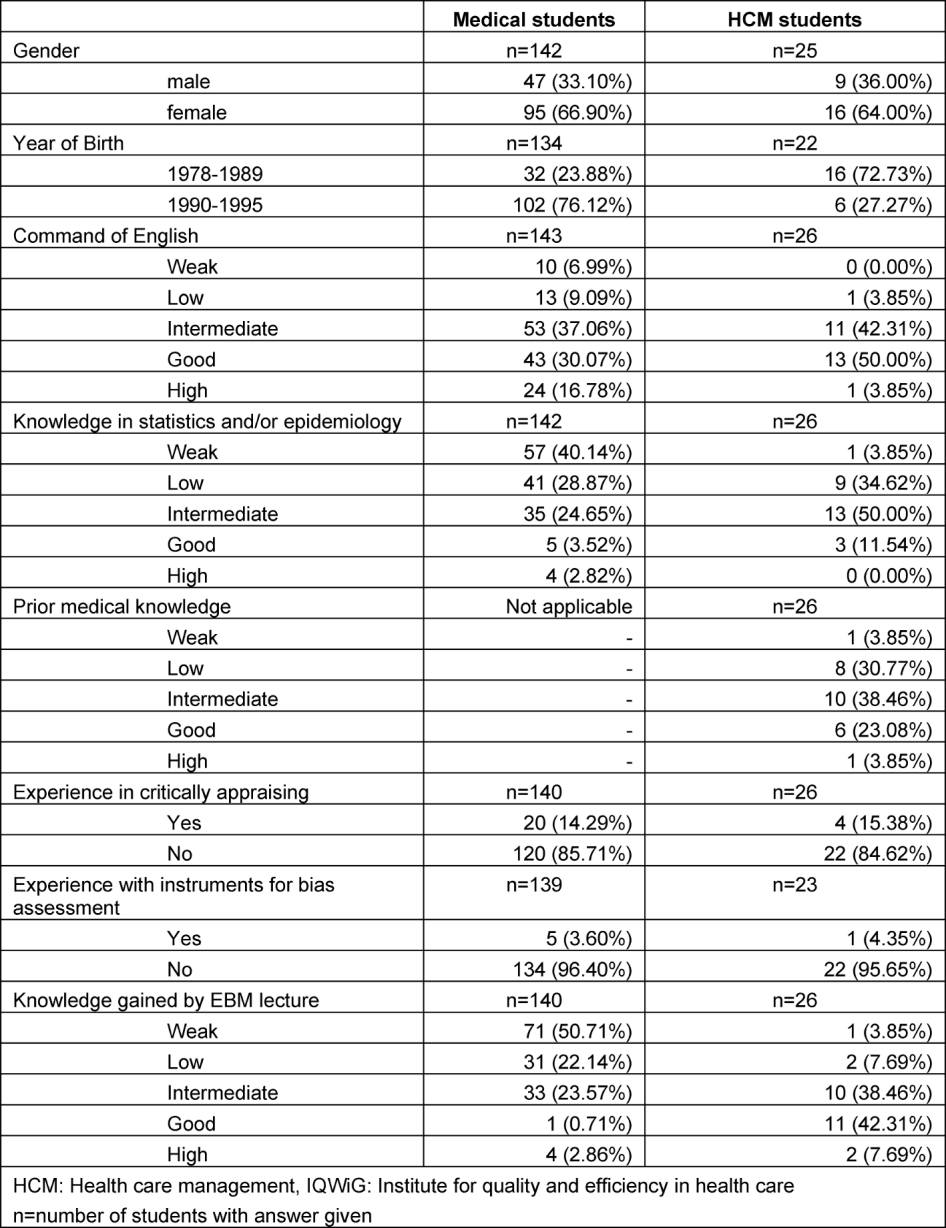
Characteristics of Students

**Table 4:**
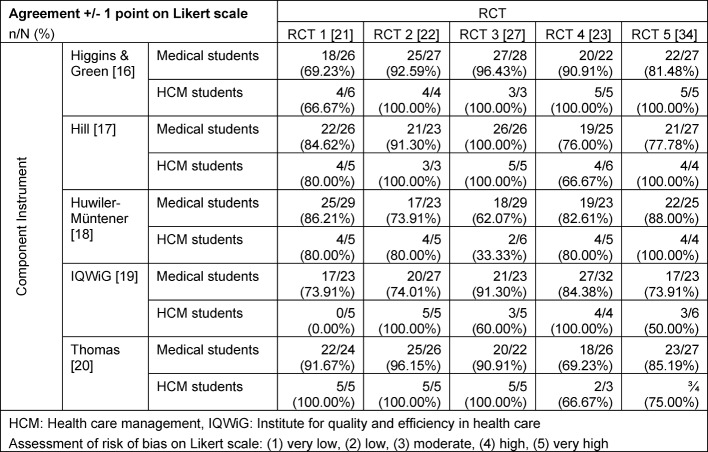
Agreement between student and expert assessments
