# Supplementary material for: Critical appraisal of RCTs by 3rd year undergraduates after short courses in EBM compared to expert appraisal
Source: GMS J Med Educ. 2018 May 15;35(2):Doc24. doi: 10.3205/zma001171 (PMC6022580; doi:10.3205/zma001171)
Supplement: Glossary of terms for quick searches [file JME-35-24-s-001.pdf]

Attachment 1: Glossary of terms for quick searches

**Term**	**Section of publication**	**Search terms**
Randomization	Abstract, Methods, Study design	random*
Concealment	Methods, Study design	sealed, opaque, envelope*, phone, centr*, blind*
Blinding	Methods, Study design	blind*, mask*, open label, unaware, independent
Drop-out/Loss to follow-up	Results	withdraw*, withdrew, drop*, lost, loss, complet*
Intention-to-treat	Methods, Study design, Statistical analysis	ITT, intention, analysis
Sample size calculation	Statistical analysis	sample size, calculation, estimat*, power, alpha
Sponsoring	First page or end of the article, Acknowledgment	grant, fund*, conflict* of interest, (consulting/speaking) fee*, support, employe*, hold stock
Bias	Discussion	bias, limit*
* Truncation, lat.: truncare: to cut		
